# Molecular mechanism of peptide editing in the tapasin–MHC I complex

**DOI:** 10.1038/srep19085

**Published:** 2016-01-12

**Authors:** Olivier Fisette, Sebastian Wingbermühle, Robert Tampé, Lars V. Schäfer

**Affiliations:** 1 Lehrstuhl für Theoretische Chemie, Ruhr-University Bochum, 44780, Germany; 2 Institute of Biochemistry, Biocenter, Goethe-University Frankfurt, 60438, Germany

## Abstract

Immune recognition of infected or malignantly transformed cells relies on antigenic peptides exposed at the cell surface by major histocompatibility complex class I (MHC I) molecules. Selection and loading of peptides onto MHC I is orchestrated by the peptide-loading complex (PLC), a multiprotein assembly whose structure has not yet been resolved. Tapasin, a central component of the PLC, stabilises MHC I and catalyses the exchange of low-affinity against high-affinity, immunodominant peptides. Up to now, the molecular basis of this peptide editing mechanism remained elusive. Here, using all-atom molecular dynamics (MD) simulations, we unravel the atomic details of how tapasin and antigen peptides act on the MHC I binding groove. Force distribution analysis reveals an intriguing molecular tug-of-war mechanism: only high-affinity peptides can exert sufficiently large forces to close the binding groove, thus overcoming the opposite forces exerted by tapasin to open it. Tapasin therefore accelerates the release of low-affinity peptides until a high-affinity antigen binds, promoting subsequent PLC break-down. Fluctuation and entropy analyses show how tapasin chaperones MHC I by stabilising it in a peptide-receptive conformation. Our results explain previous experiments and mark a key step towards a better understanding of adaptive immunity.

At the surface of all nucleated cells, major histocompatibility complex class I (MHC I) molecules (Fig. 1A,B) present peptide epitopes, resulting from cytosolic protein degradation, to cytotoxic T lymphocytes to enable the immune recognition of virally or malignantly transformed cells exposing non-self peptides^1^,^2^,^3^. Before migrating to the cell surface, MHC I molecules must first be loaded with high-affinity, immunodominant peptides. The repertoire of variable MHC I α chains is polygenic and polymorphic; each allele is highly specific to a small number of peptides. These must be selected from the pool of cytosolic degradation products containing mostly non-specific, low-affinity peptides. This selection process takes place in the endoplasmic reticulum (ER) and is mediated by the peptide-loading complex (PLC)^4^,^5^, a multiprotein assembly involving MHC I, the transporter associated with antigen processing (TAP), ERp57, calnexin or calreticulin, and tapasin (Fig. 1C). The latter acts as a hub for the assembly, recruitment, and connection of various components of the PLC. In addition, tapasin catalyses the exchange of low-affinity peptides (which, due to their abundance, are much more likely to initially be bound to MHC I) against immunodominant ones. This peptide exchange is referred to as peptide editing and is one of the key functions of the PLC^6^,^7^.

Despite its central role in the PLC, the precise working cycle of tapasin still remains largely unresolved, in particular in terms of the molecular mechanism by which tapasin stabilises MHC I and catalyses peptide exchange. The lack of high-resolution structural data for the tapasin–MHC I complex contributes to this deficiency. However, high-resolution structures of isolated MHC I and tapasin (in complex with ERp57) have been determined^8^ and molecular docking models of the complex were proposed^9^,^10^. Mutagenesis studies^8^ have shown that residues on one side of the tapasin N-terminal (TN) domain are crucial for peptide loading, indicating the approximate location of an MHC I contact site. The corresponding region on MHC I was located through mutations that abrogate binding to the PLC, leading to the current view that tapasin acts on the binding groove via the MHC I α_2_ domain^11^. Furthermore, interactions between the tapasin C-terminal domain (TC) and the CD8 recognition loop in MHC I α_3_ were also mapped out by mutagenesis^12^.

Previous simulation studies of antigen loading have focused on isolated MHC I molecules and their bound peptides rather than the MHC I–tapasin interaction. Molecular dynamics (MD) studies have compared empty (peptide-deficient, PD) and peptide-loaded (PL) MHC I^13^, highlighting the higher structural heterogeneity of the PD form. MD has also been used to investigate the differences between couples of MHC I alleles, such as tapasin-dependent *vs* -independent^14^,^15^,^16^,^17^,^18^, or normal *vs* disease-inducing^19^. Conformational changes associated with peptide loading have also been studied by MD^17^,^20^,^21^,^22^.

Previously, we characterised the encounter between MHC I allele B*44:02 and human tapasin by MD simulations^23^. In the tapasin–MHC I complex, PD MHC I exhibits a wider peptide-binding groove and better surface complementarity with tapasin than PL MHC I. Since the α_2−1_ region of MHC I is cradled by tapasin TN, we concluded that tapasin acts on MHC I by widening the peptide-binding groove, thereby accelerating the release kinetics of low-affinity peptides. Thus, tapasin and the antigen peptide (Ag) compete for the α_2−1_ region of MHC I and respectively open and close the binding groove, like two players of a molecular tug-of-war mechanism^23^. Verifying this proposed mechanism requires a detailed, spatially resolved analysis of the forces exerted on the rope (MHC I α_2−1_) by the players (tapasin and Ag). Here, based on additional multi-microsecond MD simulations, we unravel the driving forces underlying this mechanism. A detailed analysis of inter-residue forces shows that a tapasin loop bearing R187 and the peptide C-terminus are the major players of this open/close competition, explaining previous mutagenesis experiments. Computed entropy estimates illustrate the effect of peptide-loading on the thermodynamic stability of the complex. Entropy differences, observed in both tapasin and MHC I, substantiate the measured higher affinity of tapasin for peptide-deficient MHC I. Comparative simulations of PL and PD MHC I, both in complex with tapasin and in the free form, also reveal the molecular basis for the chaperone function of tapasin on MHC I. Additional simulations of isolated tapasin show that the tapasin–MHC I complex is formed by a conformational selection mechanism. The large-scale rotation of tapasin TN with respect to the TC domain, which is required for MHC I binding, is already observed in isolated tapasin, also explaining why established protein-protein docking methods were unsuccessful in predicting the structure of the complex. Taken together, our results provide a detailed atomic-level picture of the mechanisms by which MHC I is stabilised and how selection of antigen peptides is facilitated.

## Results

### Structure of the tapasin-MHC I complex

In a first attempt to predict the structure of the complex, we used molecular docking. Since MHC I α_2_ T134 and TN R187 are both known to be essential for complex assembly^8^,^24^, we used local Rosetta docking with a half-harmonic flat-bottom distance restraint acting beyond 20 Å to keep these two residues in close proximity. Given the importance of α_3_ E222 for MHC I recruitment by tapasin^12^, a second restraint was used to keep this residue in proximity to the TC domain. After filtering and clustering, a consensus structure was obtained (Fig. 2A) that matched the expected global features of the complex: two distinct interfaces, with the MHC I α_2−1_ helix of the binding groove and the CD8 recognition loop both contacting tapasin.

To validate this complex structure predicted from Rosetta docking, we used it as a starting structure for a series of all-atom MD simulations. If the specific interactions between the two proteins were correctly described, we would expect the complex to be stable on the accessible µs timescale. However, we repeatedly observed that the α_3_ -TC interface is disrupted during the simulations (Fig. 2B). While the overall orientation of the two partners may be roughly correct, the specific contacts required to form a stable complex are not successfully predicted by docking. One docking model from the literature^9^ proposes that the C-terminal domains are stabilised through a salt bridge between α_2_ E222 and TC R333; this structural feature is also found in our docking results but is not sufficient for complex stability, as our MD simulations show. Another model^10^ proposes slightly different contacts, still involving the E222–R333 pair, in addition to tapasin H334 and H335, and α3 D227. In our docking model, tapasin H334 and H335 are hydrogen-bonded to MHC I (although α_3_ D227 is not involved), but again this does not sufficiently stabilise the complex.

In light of these results, we next turned to all-atom MD simulations in explicit solvent to obtain a stable structure of the tapasin–MHC I complex. Although computationally much more demanding, this approach does, in principle, not require any prior knowledge of the bound structure. In addition, flexibility of the binding partners and explicit solvation effects are fully taken into account. We initiated our MD simulations from the crystal structures, only using the rough relative orientation of the molecules as determined by molecular docking and known from experiments. The two proteins were separated by water (Fig. 2C), and the simulations were allowed to proceed in an unbiased way, i.e. without any additional external potentials to enforce complex formation. Previously, we reported on a single successful complex formation simulation, which was initiated from the peptide-loaded MHC I state^23^. Here, we report results from 40 MD simulations. In total, 20 simulations were started from the peptide-loaded state, and 20 from the peptide-deficient state. Six of these yielded similar, stable complex structures. In the remaining trajectories, the two proteins diffused away from each other or established non-productive contacts. These simulations were considered unsuccessful and thus discontinued after less than 500 ns, whereas the others were extended to 1.0 μs. The success rate may be considered low (only 6/40 MD simulations yielded a tapasin–MHC I complex), but shows that the initial configuration by itself did not introduce an unwanted bias toward complex formation. The six successful complex formation simulations converged to similar structures, as indicated by a mean pairwise C_α_ RMSD of 4.4 Å (min 2.4 Å, max 5.6 Å) after 1.0 μs of MD. This RMSD is considered low in light of the size of the complex (756 residues) and the substantial simulation times. This reproducibility, in addition to the observed stability of the complex and the agreement with the available experimental data, strongly validates the obtained structures.

**Figure 1:**
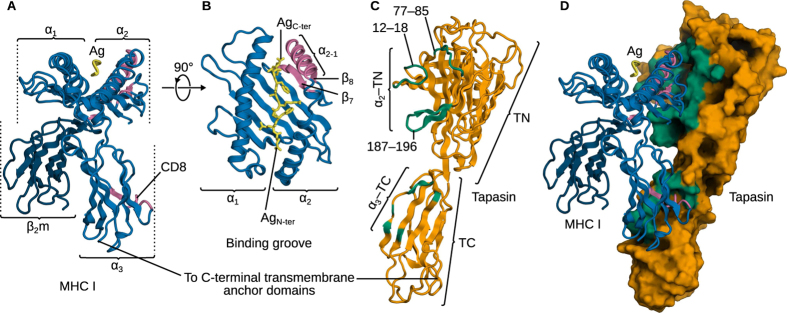
Structures of MHC I and tapasin. (**A**) MHC I contains a heavy, highly variable chain and an invariant, light β_2_m chain. The α_3_ domain bears the loop for CD8 recognition at the cell surface, and the α_1_ and α_2_ domains form the antigen peptide (Ag) binding groove (**B**). The α_2−1_ helix segment and β_7,8_ strands (at the bottom of the groove) contact three loops of the N-terminal domain (TN) of tapasin (**C**), and the CD8 loop contacts the C-terminal, Ig-like tapasin domain (TC). (**D**) Tapasin–MHC I complex obtained from our MD simulations. The two distinct N- and C-terminal interfaces are highlighted.

**Figure 2:**
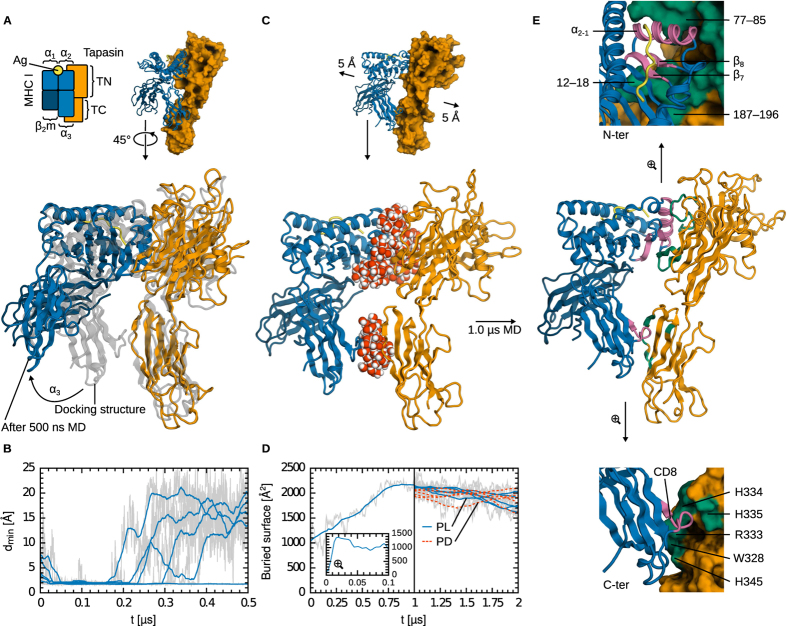
Tapasin–MHC I complex from molecular docking and MD simulations. (**A**) Prediction of the tapasin-MHC I interface from Rosetta docking yielded an unstable complex, as evidenced by the minimum α_3_ –TC distance (**B**) from 5 independent 500-ns MD simulations. (**C**) 40 MD simulations of complex formation were initiated, in which the two protein X-ray structures were separated by 10 Å and, therefore, fully separated by solvent. Six trajectories led to a productive association. (**D**) Buried surface showing spontaneous complex formation over 1 μs (example from one simulation with peptide-loaded (PL) MHC I). The structure after 1 μs was used to initiate 5 additional, independent trajectories, each recorded for 1 μs. Simulations of the peptide-deficient (PD) complex were initiated after peptide removal from the PL complex. (**E**) Tapasin–MHC I complex. Zooms on the N- and C-terminal interfaces show how MHC I α_2−1_ is cradled by tapasin, while the CD8 recognition loop contacts a cluster of basic residues. Time series in **B** and **D** are overlaid with their 1-ns moving average.

**Figure 3:**
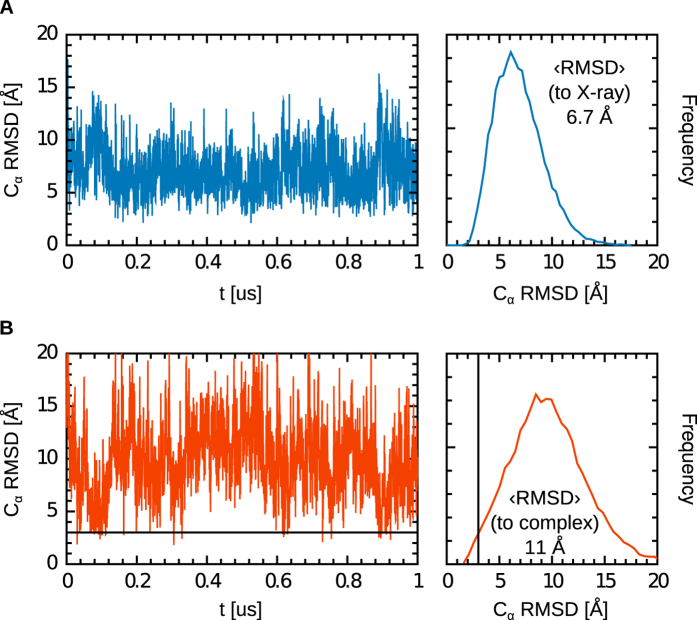
C_α_ RMSD timeseries and distribution of tapasin TC domain from a representative 1-μs simulation of free tapasin. RMSD were computed relative to the tapasin X-ray crystal structure (**A**) and to tapasin in the MHC I complex (**B**). In both cases, tapasin TN was used as the RMSD-minimising reference frame. Rotation around the TN–TC hinge leads to average TC orientations that deviate from those observed in the X-ray crystal and the complex structures. However, repeated drops below 3.0 Å (black lines in **B**) show that TC can adopt an orientation productive for MHC I complex formation (conformational selection mechanism).

The N-terminal interface predicted by docking was found to be stable in our MD simulations. This interface (Fig. 2E) involves two structural elements from the MHC I binding groove: the α_2−1_ helix fragment and the β sheet. α_2−1_ is nestled in a tapasin surface cavity bordered by two loops comprising residues 12–18 and 77–85. Strands β_7,8_ are contacted by tapasin loop 187–196. MHC I residue T134 is in close proximity to tapasin R187 in our MD simulations, transiently establishing a hydrogen bond. In addition, tapasin R187 also contacts other MHC I residues in the vicinity of T134 (N127, D129). Our simulations thus agree with the available experimental data supporting the importance of the T134/R187 pair^8^,^24^, but they also suggest that neighbouring residues are involved. The failure of protein-protein docking to recover the tapasin–MHC I C-terminal interface can likely be ascribed to the lack of a sufficient degree of protein flexibility. The tapasin N- and C-terminal domains are connected by a flexible hinge, around which we observe substantial motion in our MD simulations (Fig. 3). This plasticity allows rotation of the TN and TC domains with respect to each other, enabling contacts between the CD8 recognition loop in MHC I α_3_ (residues 222–227 and 229) and tapasin residues W328, S330 and H345, in addition to those already observed in molecular docking (H299, R333, H334, H335). Together, these contacts lead to a stable interface (Fig. 1C,D and Fig. 2E). The C-terminal Tsn residues identified here have previously been suggested to be involved in the interaction and have been shown to influence assembly and surface expression of MHC I molecules^10^,^25^,^26^,^27^.

These results, however, do not answer the question of why the MD simulations started from the docked structure never led to the formation of a stable complex, whereas those started from the fully separated structures did, even though the two proteins were initially farther apart. To address this question and to further characterise the mechanism of complex formation, we carried out three additional 1.0-μs MD simulations of isolated tapasin. These simulations confirm the observation from the complex simulations that the TC and TN domains are connected by a flexible hinge. The C_α_ RMSD of the TC domain from the X-ray crystal structure (Fig. 3A) is on average 6.7 Å (using TN as the RMSD-minimising reference frame, such that overall translation and rotation of TC with respect to TN make dominant contributions to this RMSD). For comparison, the RMSD from the complex structure is much larger, on average 11 Å (Fig. 2B). Interestingly, this RMSD repeatedly drops to a low value during the 1.0-µ s simulations. In approximately 0.5% of the recorded configurations, the RMSD is below 3.0 Å. These RMSD drops are transient (they typically last less than 2 ns) and are observed several times in each of the three trajectories, indicating a dynamic event on the hundred ns timescale. This observation strongly speaks in favour of a conformational selection-type mechanism of tapasin–MHC I complex formation. Visiting a configuration conducive to complex formation is a rare event, explaining why most simulations failed to recover the complex structure, including those started from the molecular docking structure. In addition, formation of the N-terminal interface could decrease the flexibility around the TN-TC hinge, hampering the conformational changes necessary to visit an on-pathway configuration for the formation of the C-terminal interface. Resolving this latter issue is beyond the scope of the present work. A first step in that direction could be to compare the flexibility of the TN-TC hinge in isolated tapasin and in tapasin complexed to a truncated MHC I molecule with only the α_1_ and α_2_ domains.

### Differential binding of tapasin to MHC I in the PD/PL forms

**Figure 4:**
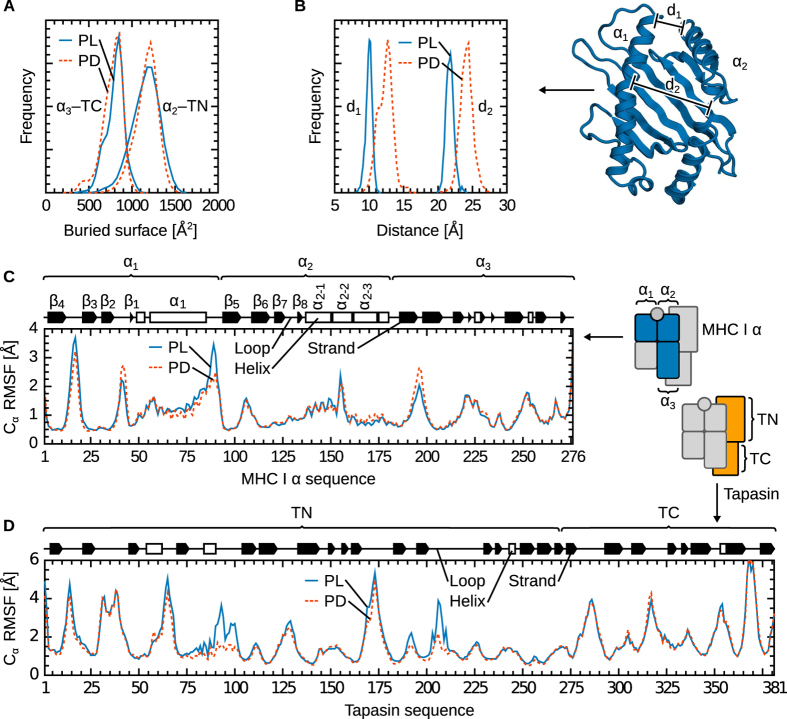
Comparison of the peptide-deficient and -loaded tapasin–MHC I complexes. (**A**) Distribution of the buried surface for the PD and PL states is similar for both the α_2_–TN and α_3_–TC interfaces. (**B**) In the absence of peptide, the binding groove widens by about 2 Å. The C_α_–C_α_ distances d1 (I85–T138) and d2 (Y74–A149) measure the width of the groove in the F-pocket region and at the centre, respectively. The widening is not linked to a significant increase in backbone flexibility at the individual residue level, as evidenced by largely unchanged C_α_ RMSF in MHC I (**C**) and tapasin (**D**).

Next, we used the structure of the tapasin–MHC I complex to initiate comparative simulations of tapasin bound to the PD and PL forms of MHC I (Fig. 2D). The peptide used in our simulations is a specific, high-affinity antigen exposed by MHC I allele B*44:02. Accordingly, we expect our results to be transferable to other tapasin-dependent MHC I alleles in complex with their specific peptides. Our main objective was to understand the molecular basis of the higher affinity of tapasin for PD MHC I and, therefore, the mechanism of its peptide-loading activity. Fig. 4A shows that the buried surface is similar in the PD and PL forms. Conformational changes are localised to the peptide-binding groove, which widens by about 2 Å in the PD form (Fig. 4B). Peptide-loaded MHC I is thus slightly more compact due to a network of contacts between the peptide and the binding groove, particularly at the Ag C-terminus in the MHC I F-pocket. The observation from previous MD simulations that isolated MHC I in the PD form exhibits higher fluctuations^13^ is consistent with this widening, which is likely necessary to ease peptide entry into the binding groove. Surprisingly, however, this conformational change has little impact on backbone flexibility at the individual residue level: C_α_ RMS fluctuations along the MHC I sequence are similar in the PD and PL forms (Fig. 4C). This behaviour differs from that of MHC I in the absence of tapasin^13^. However, as discussed below, it is compatible with a chaperone function of tapasin on MHC I. Tapasin RMSF are also largely unaffected by peptide binding, with the exception of a slight increase in fluctuations of residues 90–100 and 200–210 in the PL form (Fig. 4D).

Our previous work^23^ highlighted that surface complementarity between tapasin and MHC I is better in the PD than in the PL form, leading to small occupancy differences for the most prevalent residue-residue contacts. These prior findings and the results described here (Fig. 4) suggest that peptide binding has little influence on the global conformation of the tapasin–MHC I complex. However, purely geometric analyses may overlook mechanical forces and how they are distributed over the structure and thus relate to function. Stable equilibrium dynamics imply that overall net forces acting on the complex are small, but they reveal very little about the magnitude of local forces between individual residues, which can be substantial^28^.

**Figure 5:**
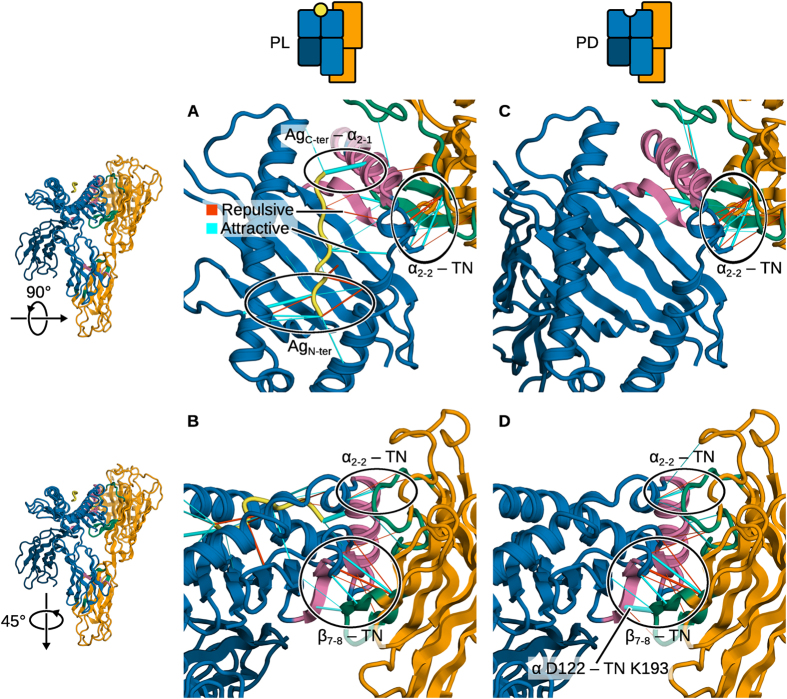
Pairwise residue-residue forces from FDA in the tapasin–MHC I complex. (**A**) In the PL complex, the antigen peptide C-terminus pulls on the α_2−1_ region to close the binding groove. The first two N-terminal Ag residues also contribute to groove stability. Tapasin acts on the α_2-2_ region. (**B**) The strongest pairwise forces between tapasin and MHC I occur on the underside (β_7,8_) of the binding groove. (**C**) In the PD complex, the same forces are seen between tapasin and MHC I. (**D**) The forces between β_7,8_ and TN are stronger in the PD than in the PL form, particularly between D122 and TN K193. Cylinders are scaled according to force magnitude. TC, α_3_, and β_2_m are not shown for clarity.

We used force distribution analysis (FDA)^28^ to analyse residue-residue forces between the complex partners in both states of the complex (PD and PL). Results are shown in Fig. 5 and the strongest forces are listed in Table 1. The forces involved in the hydrogen-bonding network at the Ag termini play a dominant role. At the F-pocket, the peptide C-terminus pulls on the α_2−1_ helix fragment and closes the groove (Fig. 5A); its absence is responsible for the widening of the cleft in PD MHC I. However, motions in α_2−1_ are restricted by the presence of tapasin, which cradles the helix and prevents it from partially dissociating from the binding groove.

One could intuitively expect tapasin to pull in turn on the α_2−1_ region to widen the binding groove and thereby facilitate peptide exchange. However, interactions between tapasin and MHC I take place mostly at the β sheet forming the floor of the groove (Fig. 5B,D). There, tapasin pulls on β_7,8_. Since these strands support the α_2_ helix, the resulting effect is the same: promoting the opening of the binding groove. Simulations of free MHC I (see next section) show that a large displacement of α_2−1_ is associated with complete dissociation of β_8_ and partial dissociation of β_7_. Interestingly, a single-point mutation (D116Y) in the floor of the binding groove converts B*44:02 to tapasin-independent allele B*44:05. The observed pull from underneath the strand is also consistent with mutagenesis data. Variant proteins TN6 (E185K, R187E, Q189S, Q261S) and TN7 (H190S, L191A, K193E) have reduced *in vitro* activity (8% and 53% compared to the wild-type enzyme, respectively)^8^. Although limited spatial resolution and the difficulty to directly measure forces between individual residues make it very challenging to unequivocally prove our tug-of-war mechanism via experiments, this agreement provides another strong validation of our simulations. Here, we assign R187 and K193 as the main contributors to the attractive pairwise forces. Given the critical importance of R187 and K193 for peptide loading, and considering that their position in the complex prevents them from interacting directly with α_2−1_ or the peptide in the binding groove, it is clear that peptide exchange is most efficiently promoted by destabilising the floor of the groove rather than any other MHC I region.

The strongest attractive pairwise force (between tapasin K193 and MHC I D122) decreases almost two-fold upon peptide loading (Δ*F* = 234 pN, Table 1). This further demonstrates that tapasin acts from underneath the β sheet and is consistent with the lower tapasin affinity for PL than for PD MHC I. This lowered affinity promotes the breakdown of the tapasin–MHC I complex and, ultimately, of the entire PLC once a high-affinity Ag peptide has been loaded onto MHC I. The location of these forces exerted by tapasin (at the F-pocket, near the C-terminus of the peptide) also agrees with recent reciprocal immunisation experiments using tapasin- and ERAAP-deficient mice, which showed that tapasin edits peptides at their C-terminus while the ER-resident aminopeptidase ERAAP performs N-terminal editing^29^. Recent MD simulation studies of H-2Kb also identified the F-pocket as a determinant of MHC I stability^30^.

A cluster of pairwise forces is also observed between MHC I α2–2 and a solvent-exposed loop in tapasin TN (Fig. 5A–D). Their magnitude is lower (< 200 pN) than for the residues involved in the attack of the β sheet described previously. We propose that they are involved in the chaperone function of tapasin. As shown in the next section, α_2–2_ acts as a flexible hinge. Unfolding of this region allows α_2−1_ to dislocate from the rest of the binding groove and move towards the solvent. Forces applied by tapasin on that region could favour a helical conformation and reduce α_2−1_ mobility.

Taken together, our results show that two opposite processes compete in the tapasin–MHC I complex. In absence of a peptide, or when only a low-affinity one is bound, tapasin widens the MHC I binding groove by pulling on its β sheet floor, displacing the α_2−1_ helix fragment and promoting peptide release. By contrast, a high-affinity peptide closes the groove by pulling directly on α_2−1_, lowering tapasin affinity for MHC I and promoting its release. The existence of these opposing forces does not, however, exclude the possibility that long-range, allosteric effects are also at play, as has been proposed^31^,^32^,^33^. A model suggesting that the interaction between tapasin TC and MHC I α_3_ relays information to the binding groove has been proposed on the basis of a computational systems biology approach^31^,^33^. In chicken, position 220 in the single, dominantly expressed MHC I locus strongly influences tapasin activity^32^. Such long-range effects could modulate MHC I structure and dynamics, and thereby possibly also influence the binding groove.

**Figure 6:**
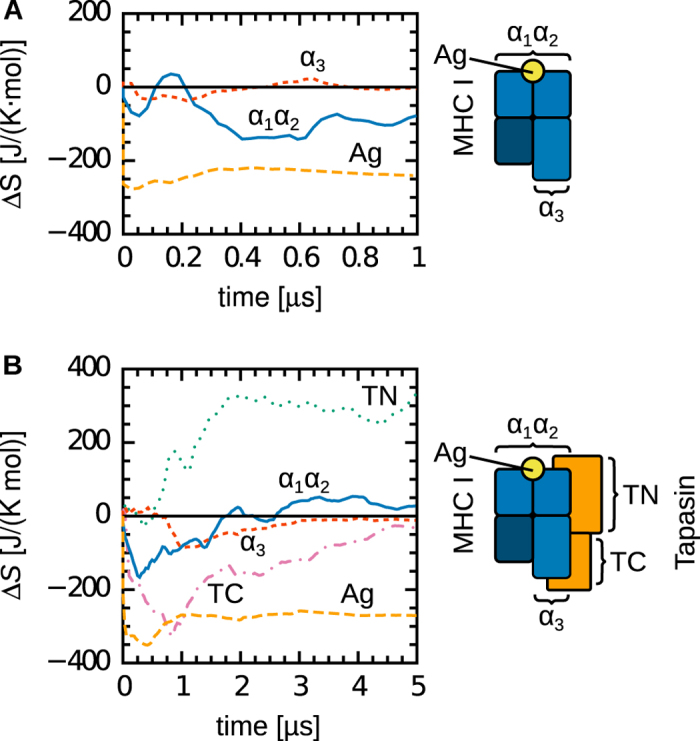
Configurational entropy changes associated with peptide loading onto free and tapasin-complexed MHC I. 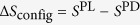
. (**A**) Association of free MHC I with a high-affinity peptide reduces the entropy of the peptide-binding groove (α_1_ and α_2_ domains). (**B**) This difference is not observed in the complex with tapasin. However, tapasin complexed with MHC I shows increased entropy upon peptide binding. This entropy increase is localised in the TN domain, with no change observed in TC. Entropy changes in the α_3_ and β_2_m domains are negligible in both forms. The entropy of the Ag peptide decreases upon binding.

If our model of a competition of forces promoting either peptide or tapasin release (depending on peptide affinity) holds, tapasin in complex with PL MHC I should exhibit higher configurational entropy than in complex with PD MHC I. The reduced interface complementarity^23^ and the lower magnitude of the pairwise forces between TN and MHC I α (Table 1) observed upon peptide loading would increase motions in tapasin as it is primed to be released from the complex. Conversely, in free MHC I, peptide loading should reduce configurational entropy due to the structuring effect of the peptide on the binding groove region. To test these hypotheses, we calculated configurational entropies^34^ from our MD trajectories. Indeed, the entropy changes associated with peptide loading support our claims (Fig. 6, Table 2). Entropy differences are localised to the N-terminal domains of both proteins (MHC I α_1_ and α_2_, tapasin TN). This was expected, given that both FDA (Fig. 5, Table 1) and contact matrix analysis^23^ showed differences between the PD and PL forms only for the N-terminal contacts between the two proteins. We observed two major effects. First, while the expected entropy decrease (−211 J/(K mol)) is observed in free MHC I upon binding of a high-affinity peptide, no significant change is seen in tapasin-complexed MHC I. This indicates again a chaperone effect of tapasin on MHC I. Second, in the complex, the entropy of the tapasin TN domain increases upon peptide binding (by +330 J/(K mol)). This localised increase could contribute to priming tapasin for dissociation from the antigen-loaded MHC I, which might be the first of a cascade of steps on the way to the break-down of the entire PLC. Interestingly, configurational entropy differences between the PD and PL states of the complex can be pronounced, despite their largely similar RMSF profiles (Fig. 4C,D). These differences only become evident when considering collective motions, as contained in the full covariance matrix of atomic fluctuations, instead of focusing only on local fluctuations of individual residues.

Up to now, we did not consider changes in configurational entropy of the peptide itself upon binding to MHC I. When bound to the complex, the antigen peptide adopts an extended conformation, resulting in a −270 J/(K mol) decrease in configurational entropy as compared to the free peptide in solution. This entropy decrease is thus similar in magnitude to the entropy increase of tapasin, such that overall, 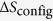 is close to zero (−10 J/(K mol)). However, one could expect peptide binding to also involve a favourable increase in solvent entropy due to a reduced solvent-accessible surface. To assess the entropy contribution of the solvent, we used an empirical relationship^35^ based on changes in solvent accessible surface. The total entropy change associated with the process is the sum of the configurational and solvent entropy changes, 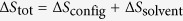. The estimated 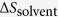 associated with peptide binding to MHC I is about 564 J/(K mol). Thus, the total entropy change estimated for peptide binding to the complex is 554 J/(K mol). This positive entropy change contributes to the overall favourable binding (ΔG < 0). Experimental studies have also shown that entropy contributes to MHC I stability^36^,^37^. In particular, peptide binding has been correlated to an increase in total entropy^36^, consistent with our observation that favourable solvent entropy changes overcome the loss of configurational entropy upon binding.

### Tapasin as MHC I chaperone

To better comprehend the molecular basis of the tapasin chaperone activity, we performed additional simulations of isolated (i.e. tapasin-free) MHC I in both the PD and PL forms. Our objectives were to identify the conformational changes that lead to MHC I molecules that are non-receptive for peptide loading, and to understand how tapasin can prevent these changes. Configurational entropy (Fig. 6) shows that the effects of tapasin are limited to the peptide-binding domain. Furthermore, the structure of the complex points to the α_2−1_ helix fragment that is cradled by tapasin (Fig. 1). FDA in turn suggests that α_2-2_ could act as a flexible hinge to favour α_2−1_ displacement (Fig. 5), a motion that would be prevented by tapasin in the complex.

**Figure 7:**
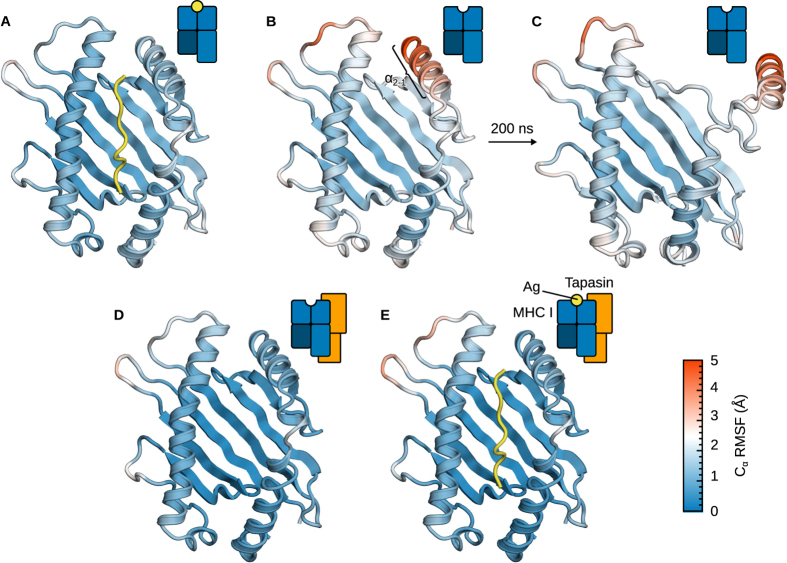
Effects of peptide and tapasin binding on MHC I fluctuations. (**A**) In its peptide-loaded, tapasin-free form, MHC I exhibits low Cα RMSF (1.0 Å median). (**B**) By contrast, in the peptide-deficient form, it shows markedly increased fluctuations, especially in the α_2−1_ region (3–5 Å C_α_ RMSF). (**C**) This is caused by a partial unfolding of the α_2_ domain, with α_2−1_ detaching from the binding groove (structure after 200 ns of MD). (**D**) Association with tapasin reduces MHC I fluctuations to a similar extent as binding of a high-affinity peptide does. (**E**) Peptide binding to tapasin-complexed MHC I does not have any additional effect. The α_3_ and β_2_m domains are not shown for clarity.

Figure 7 shows the results of three 1-μs MD simulations of free MHC I in both the PD and PL forms. As expected, peptide removal increases the fluctuations in MHC I (Fig. 7A,B). This increase is localised in the α_2−1_ region: the helix fragment is displaced from the protein, a motion that is facilitated by partial unfolding of α_2-2_ (Fig. 7C). This conformational transition is not possible in the complex since tapasin contacts MHC I on the α_2−1_ side and therefore confines it to the vicinity of the binding groove (Fig. 7D). Interestingly, addition of a high-affinity peptide to the complex does not further reduce MHC I fluctuations (Fig. 7E). This is consistent with the small entropy difference associated with peptide loading in tapasin-complexed MHC I (Fig. 6). By preventing disruption of the binding groove through contacts with the α_2−1_ and α_2-2_ regions, tapasin has a structuring effect on MHC I similar to that of a high-affinity peptide.

## Discussion

In this work, we used all-atom molecular dynamics simulations in explicit solvent to study the formation of the tapasin–MHC I complex and the mechanism by which tapasin accelerates the exchange of low-affinity against high-affinity peptides (peptide editing). Our simulations show that the tapasin–MHC I complex is formed via a conformational selection mechanism that involves structural flexibility in the uncomplexed state, explaining the failure of established protein-protein docking protocols in predicting the structure of the complex. Force distribution analysis reveals a molecular tug-of-war mechanism underlying peptide editing in tapasin-dependent MHC I alleles. Tapasin and antigen peptide both exert forces on the MHC I binding groove and respectively try to open and close it. The outward-pulling forces due to tapasin are counteracted by the inward-pulling forces due to the binding of a high-affinity peptide. Configurational entropy analysis shows that, in the peptide-deficient state, tapasin stabilises MHC I in a peptide-receptive conformation by acting on the α_1_ and α_2_ domains that form the binding groove. Upon peptide loading, the entropy of the N-terminal tapasin domain is increased, which links to its reduced affinity for MHC I. Additional simulations show that tapasin acts on the α_2−1_ region of MHC I by preventing its dissociation from the rest of the binding groove.

**Figure 8:**
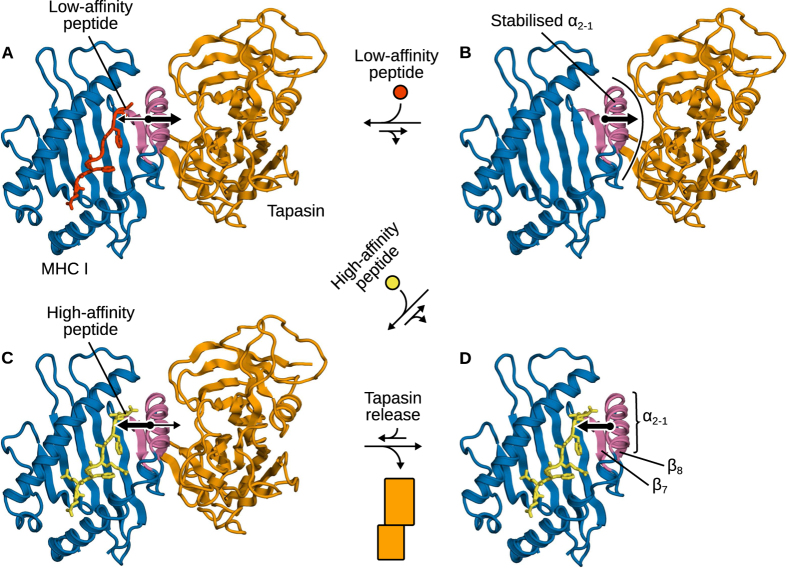
The molecular mechanism of tapasin as catalyst and chaperone. (**A**) In MHC I loaded with a low-affinity peptide, forces exerted by tapasin to widen the binding groove dominate, accelerating the kinetics of peptide release. (**B**) Peptide-deficient MHC I is stabilised in a receptive conformation by the chaperoning of tapasin. (**C**) Upon binding of a high-affinity peptide, forces exerted by the peptide to close the groove dominate, promoting tapasin release (**D**).

Taken together, these results lead to the working cycle of tapasin as both catalyst and chaperone proposed in Fig. 8. Peptide editing results from an equilibrium of forces (acting in opposite directions) exerted by tapasin and the peptide on the MHC I binding groove. When a low-affinity peptide is bound to the groove (Fig. 8A), forces from tapasin pulling the strands underneath α_2−1_ dominate. This widens the binding groove and accelerates peptide release, which is the rate-limiting step. The resulting peptide-deficient MHC I is thermodynamically stabilised (since tapasin binds peptide-deficient MHC I more strongly than MHC I loaded with a high-affinity peptide^23^) and also structurally protected from partial unfolding by direct contacts between α_2−1_ and tapasin (Fig. 8B). Upon binding of a high-affinity peptide, the forces exerted by the latter to close the groove dominate (Fig. 8C). This tightens the binding groove and decreases tapasin affinity for MHC I, priming the complex for dissociation (Fig. 8D). Peptide-loaded MHC I can then migrate to the cell surface and present the antigen to cytotoxic T cell receptors.

In the PLC, tapasin acts as a hub to bridge the TAP transporter (the peptide donor) and MHC I (the peptide acceptor). Although tapasin is known to be active in peptide editing outside its native, ER-anchored environment^8^,^23^, the presence of a membrane could influence the global organisation of the PLC, which remains to be elucidated. Furthermore, accessory proteins that merely play a structuring role, such as ERp57 and calreticulin, could also impose spatial restraints. In light of these open questions, we expect that the present work will foster future studies of the entire PLC, of which the tapasin–MHC I complex is a central component.

## Methods

### Initial structures

Peptide-loaded MHC I coordinates were taken from an X-ray crystal structure (PDB ID 1M6O)^38^ of allele B*44:02 loaded with the specific, high-affinity HLA DPA*0201 peptide. The polypeptide contains 276 residues from the MHC I heavy chain, excluding the membrane-spanning helix and cytosolic tail. Tapasin coordinates were taken from the X-ray crystal structure (PDB ID 3F8U)^8^ of the tapasin/ERp57 conjugate; chain A from the asymmetric, dimeric crystal unit was retained. Missing residue coordinates (20 residues in 4 solvent-exposed loops) were built using Modeller 9.12^39^. The resulting 381-residue protein contains the two ER-lumenal domains of tapasin and excludes its transmembrane and cytosolic regions.

### Molecular docking

Protein-protein docking was performed with RosettaDock from Rosetta 3.4^40^. Tapasin and MHC I were first aligned along their longitudinal axes as imposed by anchoring to the ER membrane. The proteins were oriented such that MHC I T134 and tapasin R187 (N-terminal domains) are proximal, and keeping E222 of MHC I close to the tapasin C-terminal domain. Local docking proceeded by random perturbation of the initial structures using Gaussians with standard deviations of 3 Å and 8° for translation and rotation, respectively. Recommended^40^ extra side chain rotamers were included. Harmonic potential energy functions acting beyond 20 Å were used to restrain the distances between T134 C_α_, R187 C_α_, and MHC I E222 C_α_ to any C_α_ in TC (tapasin residues 270–381). 10 000 candidate complex structures were generated, clustered, and finally assessed through their interface score and RMSD. The 10 best-scoring structures converged to a single cluster with low interface RMSD.

### MD simulations

Simulations were carried out with GROMACS 4.6.5^41^. The Amber99SB-ILDN protein forcefield^42^,^43^ and TIP3P^44^ water model were used. The SETTLE^45^ and LINCS^46^ constraint algorithms were applied to constrain the internal degrees of freedom of water molecules and the bonds in other molecules, respectively. In combination with virtual site hydrogens^47^, this allowed for a 4-fs integration time step. Short-range non-bonded Coulomb and Lennard-Jones 6–12 interactions were treated with a Verlet buffered pair list^48^ with potentials smoothly shifted to zero at a 10 Å cut-off. Long-range Coulomb interactions were treated with the PME method^49^ with a grid spacing of 1.2 Å and cubic spline interpolation. Analytical dispersion corrections were applied for energy and pressure to compensate for the truncation of the Lennard-Jones interactions. Periodic rhombic dodecahedron cells were used. The thermodynamic ensemble was nPT. Temperature was kept constant at 300 K by a velocity-rescaling thermostat^50^ with coupling time constant 0.1 ps. For constant 1.0 bar pressure, an isotropic Berendsen barostat^51^ was used with coupling time constant 0.5 ps and 4.5 × 10^−5^ bar^−1^ compressibility. Coordinates were saved every 20 ps.

Molecular docking validation MD simulations were started from the best-scoring Rosetta-predicted tapasin–MHC I complex structure. The system was solvated, and randomly picked water molecules were replaced by Na^+^ and Cl^−^ ions to yield a concentration of 0.15 M and a neutral overall charge. The final system contained ca. 150 000 atoms. After 500 steps of steepest-descent (SD) energy minimisation, initial velocities were generated at 65 K and the system was linearly heated up to 300 K over 1.0 ns. Five independent 500-ns trajectories were then acquired.

In contrast to the previous docking validation MD simulations, spontaneous complex formation MD simulations were initiated from the X-ray crystal structures. The two proteins were overlaid on the docked complex structure and their centres of mass were then separated by 10 Å, such that the two proteins were completely solvent-separated (Fig. 2). The PD complex was obtained by peptide removal. Although there is no X-ray crystal structure of peptide-free MHC I available, we assume that tapasin keeps MHC I in a stable, peptide-receptive conformation that is likely similar to the peptide-bound state. The structures were energy-minimised by 500 SD steps, solvated, and equilibrated, as described before. 40 independent trajectories were then initiated (20 for both the PL and PD systems). These were interrupted when the two components diffused away from each other or established non-productive contacts, or extended up to 1.0 μs when complex formation was successful.

Comparative simulations of the PL and PD forms proceeded from the final coordinates after 1.0 μs of one of the complex-forming trajectories initiated from the PL state. The PD state was prepared by replacing the peptide with water, followed by 500 steps of SD energy minimisation. Ten 1.0-μs trajectories when then acquired, five each for the PL and PD systems.

Simulations of isolated MHC I, isolated tapasin, and isolated peptide proceeded from the above-described initial structures. PD MHC I was prepared by peptide removal. The structures were energy-minimised, solvated, and equilibrated as described above. Final system size was ca. 58 000 atoms for MHC I, 132 000 for tapasin, and 8000 for the peptide. Simulation time was 5 × 200 ns for both the PL and PD forms of MHC I; 3 × 10 μs for tapasin; 10 × 500 ns for the peptide.

### Analysis

Equilibrium properties were computed from the final 90% of each trajectory. Buried surface was computed as the difference in solvent-accessible surface (SAS) between the protein complex and its components, using a probe radius of 1.4 Å. Force distribution analysis was performed using the PF2 code^28^ as implemented in GROMACS. For analysing pairwise forces between residues, the protein was divided into two groups: the first group contained tapasin TN and the peptide, the second group contained MHC I α_1_ and α_2_. Errors were estimated from block averaging^52^.

To calculate configurational entropies, we used the quasi-harmonic approximation (QHA) as formulated by Schlitter^34^,


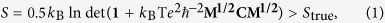


which provides an upper bound to the true configurational entropy. In this equation, 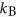 is the Boltzmann constant, T the temperature, *e* Euler’s number, ħ the reduced Planck constant, and **M** the 3N-dimensional diagonal mass matrix for the N particles. The matrix **C** is the covariance matrix of particle fluctuations,


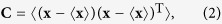


where the 3N-dimensional vector **x** represents the Cartesian coordinates of the N particles for which the entropy is calculated after removing overall translation and rotation by fitting to a reference structure. The starting structure of our simulations was used as the reference structure for this fit. The coordinates of the C_α_ atoms were used to construct **C**.

To rationalise entropy differences between the different states, the covariance analysis was not only carried out for the entire complex, but also for the individual domains separately. Although this decomposition into component contributions neglects intermolecular correlations (i.e. the entropy of the complex is not equal to the sum of the entropies of the individual components), it enables to assign entropy changes to certain structural elements.

The above approach has two principal limitations. First, QHA overestimates the true entropy due to neglect of mode anharmonicities and correlations. However, this might not be a major issue here, because we are not interested in absolute entropies, but rather in entropy differences, e.g. between isolated MHC I molecules and those that are in complex with tapasin. We assume that correlations are similar in these two states and thus largely cancel out. The second limitation is that quasi-harmonic analysis is not suitable for the solvent. Thus, to estimate the contributions due to changes in the entropy of the solvent, we used an empirical relationship between changes in polar/apolar SAS and solvent entropy^35^, 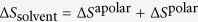, with


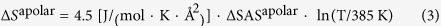


and


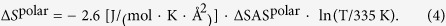


This empirical relationship cannot be expected to yield a quantitatively accurate description. Rather, it provides a rough, qualitative estimate that is useful for mechanistic interpretations, which is one of the main goals of the present study.

## Additional Information

**How to cite this article**: Fisette, O. *et al.* Molecular mechanism of peptide editing in the tapasin-MHC I complex. *Sci. Rep.* **6**, 19085; doi: 10.1038/srep19085 (2016).

## Figures and Tables

**Table 1:** Strongest residue-residue forces in the tapasin–MHC I complex.

Tapasin	Ag	MHC Iα	*F*_PD_	*F*_PL_	Δ*F*_PD→PL_
—	—	—	[pN]	[pN]	[pN]
R187	—	N127	267 ± 4	267 ± 31	0 ± 35
R187	—	D129	−295 ± 11	−312 ± 10	−17 ± 21
K193	—	D122	−532 ± 22	−298 ± 38	234 ± 60
H195	—	E128	−217 ± 8	−220 ± 15	−3 ± 23
—	E1	E63	—	−225 ± 18	—
—	E1	Y159	—	237 ± 10	—
—	E2	K45	—	−443 ± 44	—
—	F3	Y99	—	286 ± 29	—
—	F3	Y159	—	268 ± 49	—
—	R5	D156	—	−264 ± 43	—
—	F9	K146	—	−634 ± 36	—

Only forces 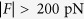 are listed. Negative forces are attractive.

**Table 2:** Configurational entropy changes associated with peptide loading onto free and tapasin-complexed MHC I.

Domain	ΔS_config_	Uncertainty
—	[J/(K mol)]	[J/(K mol)]
Free MHC I	−211	53
α1α2	−78	25
α3	−4.5	0.4
Ag	−240	2.5
Complex	−10	80
α1α2	27	3.6
α3	−11	1.1
TN	330	67
TC	−29	1.3
Ag	−270	1.4

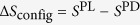
. The statistical uncertainty

was estimated from the difference between the

final 10% of cumulative sampling, 
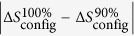
.
